# Survival prediction using temporal muscle thickness measurements on cranial magnetic resonance images in patients with newly diagnosed brain metastases

**DOI:** 10.1007/s00330-016-4707-6

**Published:** 2017-01-03

**Authors:** Julia Furtner, Anna S. Berghoff, Omar M. Albtoush, Ramona Woitek, Ulrika Asenbaum, Daniela Prayer, Georg Widhalm, Brigitte Gatterbauer, Karin Dieckmann, Peter Birner, Bernadette Aretin, Rupert Bartsch, Christoph C. Zielinski, Veronika Schöpf, Matthias Preusser

**Affiliations:** 1 Department of Biomedical Imaging and Image-guided Therapy, Medical University of Vienna, Vienna, Austria; 2 Comprehensive Cancer Center, Central Nervous System Tumor Unit (CCC-CNS), Medical University of Vienna, Vienna, Austria; 3 Department of Medicine I, Medical University of Vienna, Vienna, Austria; 4 Department of Radiology and Nuclear Medicine, University of Jordan, Amman, Jordan; 5 Department of Neurosurgery, Medical University of Vienna, Vienna, Austria; 6 Department of Radiotherapy, Medical University of Vienna, Vienna, Austria; 7 Department of Pathology, Medical University of Vienna, Vienna, Austria; 8 Pharmacy Department, General Hospital Vienna, Vienna, Austria; 9 Institute of Psychology, University of Graz, Graz, Austria; 10 BioTechMed, Graz, Austria

**Keywords:** Brain metastases, Sarcopenia, Prognosis, Diagnosis-specific graded prognostic assessment, Cancer cachexia

## Abstract

**Objectives:**

To evaluate the prognostic relevance of temporal muscle thickness (TMT) in brain metastasis patients.

**Methods:**

We retrospectively analysed TMT on magnetic resonance (MR) images at diagnosis of brain metastasis in two independent cohorts of 188 breast cancer (BC) and 247 non-small cell lung cancer (NSCLC) patients (overall: 435 patients).

**Results:**

Survival analysis using a Cox regression model showed a reduced risk of death by 19% with every additional millimetre of baseline TMT in the BC cohort and by 24% in the NSCLC cohort. Multivariate analysis included TMT and diagnosis-specific graded prognostic assessment (DS-GPA) as covariates in the BC cohort (TMT: HR 0.791/CI [0.703–0.889]/p < 0.001; DS-GPA: HR 1.433/CI [1.160–1.771]/p = 0.001), and TMT, gender and DS-GPA in the NSCLC cohort (TMT: HR 0.710/CI [0.646–0.780]/p < 0.001; gender: HR 0.516/CI [0.387–0.687]/p < 0.001; DS-GPA: HR 1.205/CI [1.018–1.426]/p = 0.030).

**Conclusion:**

TMT is easily and reproducibly assessable on routine MR images and is an independent predictor of survival in patients with newly diagnosed brain metastasis from BC and NSCLC. TMT may help to better define frail patient populations and thus facilitate patient selection for therapeutic measures or clinical trials. Further prospective studies are needed to correlate TMT with other clinical frailty parameters of patients.

***Key Points*:**

• *TMT has an independent prognostic relevance in brain metastasis patients*.

• *It is an easily and reproducibly parameter assessable on routine cranial MRI*.

• *This parameter may aid in patient selection and stratification in clinical trials*.

• *TMT may serve as surrogate marker for sarcopenia*.

**Electronic supplementary material:**

The online version of this article (doi:10.1007/s00330-016-4707-6) contains supplementary material, which is available to authorized users.

## Introduction

Brain metastases are a frequent occurrence, and affect up to 40% of cancer patients during their disease course. Primary tumour types differ in their tendency to spread to the CNS, with non-small cell lung cancer (NSCLC) and breast cancer (BC) showing the highest frequency of brain metastases in the clinical setting [1, 2]. Brain metastases are associated with a significant symptom burden, a reduced quality of life, and poor survival times. Therapy strategies include neurosurgical resection, radiation therapy (whole-brain radiotherapy or stereotactic radiosurgery) and systemic therapies depending on the number, location and size of CNS lesions, the extracranial disease status, the patient’s clinical condition and histological and molecular tumour characteristics [3].

Brain metastases as a result of NSCLC and BC are generally considered incurable and are associated with poor median overall survival times. However, individual survival times show a high variability and range from 3 to 14.8 months in NSCLC and 3.4 to 25.3 months in BC patients with brain metastases [4, 5]. Established prognostic scores consider clinical factors, such as the number of brain metastases, the extracranial tumour status and the Karnofsky performance scale for outcome prediction. However, recently scores based solely on these clinical characteristics were shown to lack accuracy in survival prediction [6]. In particular, the assessment of the patient’s physical capacity is based on the subjective judgement of the attending physician and is prone to observer variability [7]. More objective measures of the patient’s physical condition may help to improve outcome prediction.

Cancer-related cachexia has been defined by international consensus as a syndrome that consists of skeletal muscle mass loss, which leads to progressive functional impairment without the possibility of complete reversal by conventional nutritional support [8]. In addition to weight loss, sarcopenia, which is defined as a loss of skeletal muscle mass and function, has a core role in cancer-related cachexia [8]. Due to its association with disability and functional impairment, sarcopenia can be used as an objectively measurable parameter of frailty. Recent studies revealed a high correlation between sarcopenia and reduced long-term outcome in several cancer types [9, 10]. The majority of these observations were based on skeletal muscle index cut-offs determined by the total cross-sectional skeletal muscle area at the level of the third lumbar vertebra, using computed tomography (CT) scans [9, 11]. Moreover, the cross-sectional area of the psoas muscle at the level of lumbar vertebra L3 has also been determined to be a reliable marker of sarcopenia, thus increasing the practicality of the measurement in the routine clinical setting [12]. A recent study revealed a high correlation between the psoas muscle area and temporal muscle thickness (TMT), indicating that not only abdominal muscles but also craniofacial muscles are useful as indicators of patient frailty [13]. This would be advantageous, especially in patients with brain tumours, because TMT can be evaluated on routine brain CT or magnetic resonance imaging (MRI) examinations. We thus hypothesize that TMT may serve as a surrogate marker of patient frailty that can easily be measured on standard MR images in brain tumour patients. We have, therefore, investigated the prognostic relevance of TMT in two large and independent series of BC and NSCLC brain metastasis patients.

## Methods

### Patients

All patients treated for newly diagnosed brain metastases from either BC or NSCLC at the Medical University of Vienna between 2008 and 2013 were identified from a brain metastasis database (n = 501). Sixty-six patients were excluded because: (a) MRI images were not retrievable (n = 26), (b) only CT images of the brain were available (n = 31) or (c) images were inaccurate due to motion artifacts (n = 9). Clinical data, including information on the primary tumour, clinical course and survival times, were retrieved by chart review. The diagnosis-specific graded prognostic assessment (DS-GPA) is an established and validated clinical prognostic assessment based on various clinical factors. Importantly, the included clinical factors differ depending on the underlying primary tumour type. In NSCLC the DS-GPA is calculated based on age, number of brain metastasis, Karnofsky performance status and presence of extracranial metastases. In breast cancer the DS-GPA includes age, breast cancer subtype and Karnofsky performance status. DS-GPA calculation was applied as previously described by Sperduto et al. [5].The ethics committee of the Medical University of Vienna approved the study (Vote 078/2004).

### Analyses of temporal muscle thickness (TMT)

**Fig. 1:**
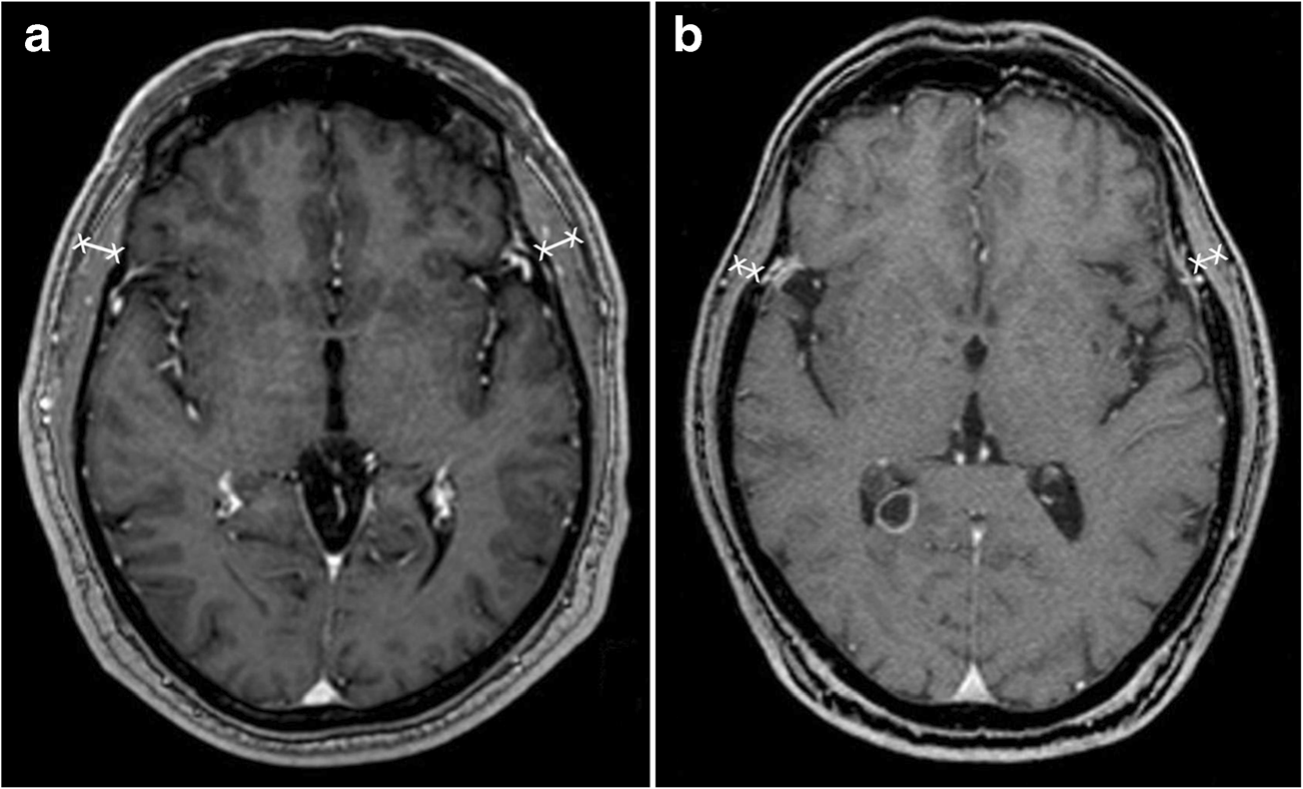
Magnetic resonance images representing temporal muscle thickness (TMT) assessment. (**A**) A 58-year-old male patient with an overall survival of 59 months (median TMT = 11.2 mm), and (**B**) a 68-year-old male patient with an overall survival of 1 month (median TMT = 4.6 mm)

Baseline TMT at diagnosis of brain metastasis was measured on the axial plane of isovoxel (1 × 1 × 1 mm) T1-weighted MR images perpendicular to the long axis of the temporal muscle at the level of the orbital roof. Axial MR plane was oriented parallel to the anterior commissure–posterior commissure line. The Sylvian fissure defined a reference point regarding the anterior-posterior orientation. These landmarks were defined before all TMT measurements to increase the accuracy of TMT measurement in each patient. In all patients, TMT on the left and on the right side were determined separately. One board-certified radiologist (JF, the main observer) analysed TMT for all included patients. For the BC data only female patients were included. Examples of TMT assessment on MR images are illustrated in Fig. 1. Both subcohorts (120/188 BC; 120/247 NSCLC) were evaluated by one additional, independent board-certified radiologist blinded to the results of the main observer and to all clinical characteristics. Intraclass correlations (ICC) were estimated to assess intra-observer reliability using a two-way mixed model for both sub-cohorts respectively. Values were interpreted as no agreement for values <0, as slight agreement for 0–0.20, as fair for 0.21–0.40, as moderate for 0.41–0.60, as substantial for 0.61–0.80, and as almost perfect agreement for 0.81–1.

### Statistical analysis

Overall survival (OS) was defined as from the diagnosis of brain metastasis to death or date of last follow-up. Median TMT was assessed by summing the measurements from each side and then dividing by two, which resulted in one variable – the median TMT per patient. TMT was grouped according to the median per group and the Kaplan Meier product was applied to retrieve survival curves. The log-rank test was used to investigate differences between groups. TMT was entered as a scale variable in a Cox regression model to investigate the association with survival times. The Spearman correlation coefficient was used to correlate two scale variables. A Spearman correlation coefficient from (-)0.7 to (-)1 was regarded as a strong association, a correlation coefficient of (-)0.5 to (-)0.7 as moderate, and a correlation coefficient of (-)0.3 to (-)0.5 as a low association. A Spearman correlation coefficient of 0 to (-)0.3 was interpreted as no association. A two-tailed p-value of <0.05 was considered statistically significant. Due to the hypothesis-generating approach of the current paper, no correlation for multiple testing was applied [14].

## Results

### Patient characteristics

Two independent cohorts consisting of 188 patients with newly diagnosed brain metastasis from BC (BC cohort) and 247 patients with newly diagnosed brain metastasis from NSCLC (NSCLC cohort) were available for further analysis. Therefore, overall, 435 patients were included in the final analysis. Patient characteristics, including clinical characteristics, survival times and applied therapies are provided as Supplemental Table 1.

### Assessment of TMT and observer agreement

The median TMT on the right side was 5.3 mm (range 1.1–10.6), and 5.4 mm (range 1.2–11.0) on the left side, resulting in a median of 5.4 mm (range 1.6–10.5) in the BC cohort. In the NSCLC cohort, the median TMT on the right side was 6.0 mm (range 2.1–11.0), and 5.8 mm (range 2.0–12.8) on the left side, resulting in a median of 5.9 mm (range 2.2–11.0) in this group.

Inter-observer reliability was assessed and revealed almost perfect agreement between both observers for NSCLC (n = 120 patients, ICC = 0.995, CI 0.993–0.996, p < 0.001) and BC (n = 120 patients, ICC = 0.996, CI 0.995–0.997, p < 0.001) sub-cohorts (Supplemental Fig. 1). Based on this almost perfect agreement between both observers in the two sub-cohorts, TMT calculations were, as described in the *Statistical analysis* section, based on the measurements of the main observer for the final analysis including 435 (188 BC, 247 NSCLC) patients.

### Correlation of TMT with clinical characteristics in the breast cancer (BC) cohort

Median TMT showed a low negative correlation with age at diagnosis of brain metastasis (Spearman correlation coefficient -0.324; p < 0.001). Further, there was no correlation between median TMT and median time from diagnosis of primary tumour to diagnosis of brain metastasis (Spearman correlation coefficient -0.043; p = 0.554), body mass index (BMI) (Spearman correlation coefficient -0.164; p = 0.265) or cortisone treatment at diagnosis of brain metastasis (p = 0.108).

### Correlation of TMT with clinical characteristics in the non-small cell lung cancer (NSCLC) cohort

Age at diagnosis of brain metastasis showed a low negative correlation with median TMT (Spearman correlation coefficient -0.271; p < 0.001). Median TMT was significantly lower in female patients (5.2 mm) compared to male patients (6.3 mm) (p < 0.001; Mann-Whitney U test). However, median TMT in the female BC and NSCLC cohort showed no significant difference (p = 0.245; t-test). As already observed in the BC cohort, there was no strong correlation between median TMT and median time from diagnosis of primary tumour to diagnosis of brain metastasis (Spearman correlation coefficient 0.008; p = 0.901), BMI (Spearman correlation coefficient 0.292; p < 0.001) or cortisone treatment (p = 0.493; Mann-Whitney U test).

### Correlation of median TMT with survival time from the diagnosis of brain metastasis in the BC cohort

**Fig. 2:**
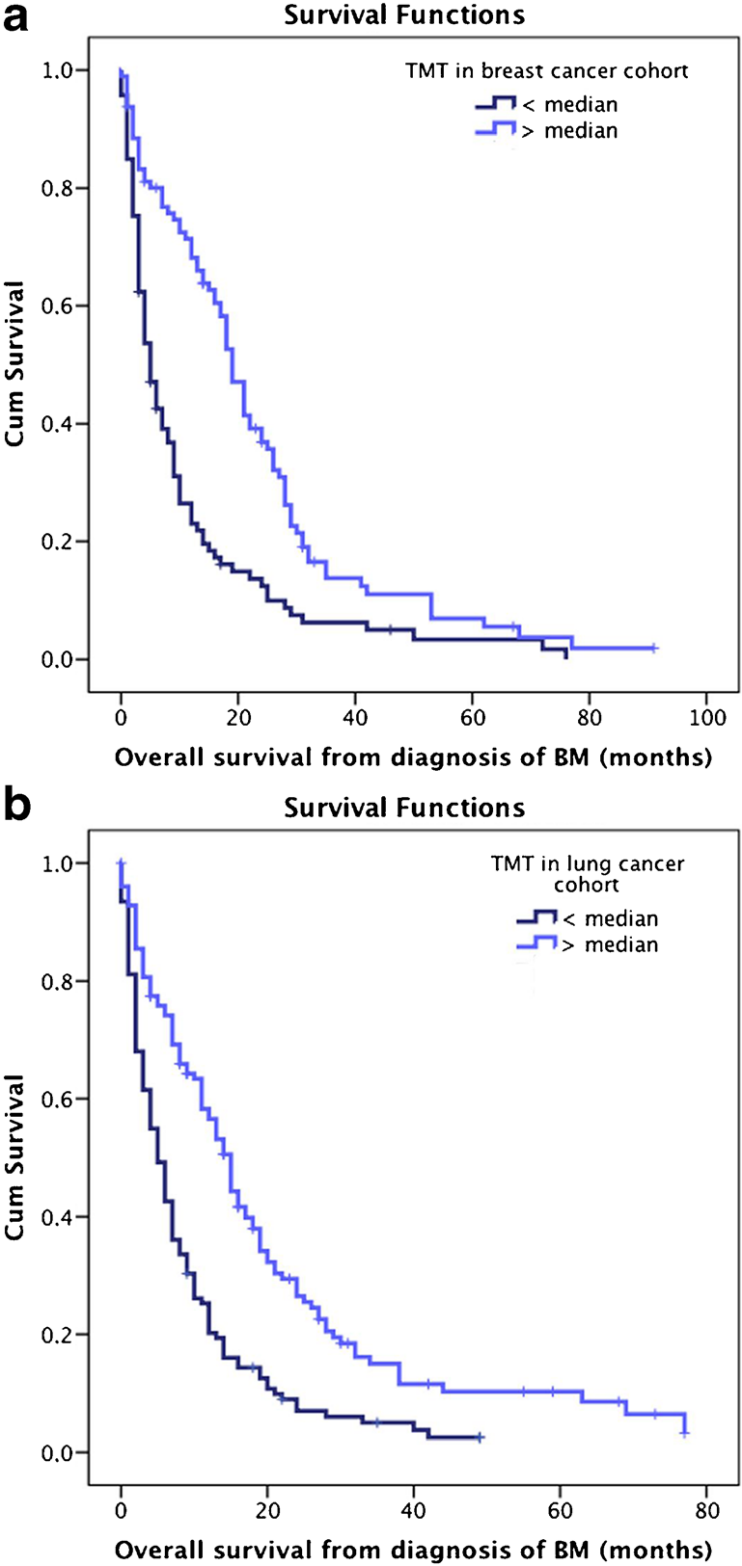
Overall survival according to median temporal muscle thickness (TMT). (**A**) Breast cancer (BC) cohort; (**B**) non-small cell lung cancer (NSCLC) cohort. *Cum* cumulative

Survival analysis, using a Cox regression model, was performed with baseline TMT diameters to predict survival time in the BC cohort. Here, patients with a higher baseline TMT had an improved survival prognosis with a hazard ratio (HR) of 0.810 (95% CI 0.736–0.892; p < 0.001; Cox regression model). Explicitly, the risk of death was reduced by 19% with every additional millimetre of baseline TMT. Patients with TMT > median (19 months) had a statistically significant longer survival time compared to patients with TMT < median (5 months; p < 0.001; log-rank test; Fig. 2A).

Further analysis was performed with a Cox regression model that included TMT and DS-GPA as covariates. In the multivariate model, TMT (HR 0.791; 95% CI 0.703–0.889; p < 0.001;) as well as DS-GPA (HR 1.433; 95% CI 1.160–1.761; p = 0.001) showed a statistically significant association with survival prognosis. Explicitly, TMT prediction of survival was nearly unchanged, with a reduced risk of death of 21% with every additional millimetre of baseline TMT.

### Correlation of TMT with survival time from the diagnosis of brain metastasis in the NSCLC cohort

A survival analysis using a Cox regression model was performed with baseline TMT diameters to predict survival time in the NSCLC cohort. Similar to the findings in the BC cohort, TMT was statistically significantly associated with survival prognosis with an HR of 0.754 (95% CI 0.692–0.821; p < 0.001; Cox regression model). Explicitly, the risk of death was reduced by 24% with every additional millimetre of baseline TMT. Patients with a TMT > median (15 months) presented with a statistically significant longer survival time compared to patients with TMT (5 months; p < 0.001; log rank test; Fig. 2B).

To assess the independence of TMT in the NSCLC cohort, further analysis was performed, using a Cox regression model that included TMT and clinical factors shown to be associated with TMT, such as gender and DS-GPA. In the multivariate model, TMT (HR 0.710; 95% CI 0.646–0.780; p < 0,001; Cox regression model) as well as gender (HR 0.516; 95% CI 0.387–0.687; p < 0.001) and DS-GPA (HR 1.205; 95% CI 1.018–1.426; p = 0.030) showed a statistically significant association with survival prognosis. Explicitly, TMT prediction of survival was stable, with a reduced risk of death of 29%, with every additional millimetre of TMT.

## Discussion

This study aimed to investigate the prognostic role of TMT measured on routinely obtained MR images of the brain in patients with brain metastases. We selected clearly defined patient cohorts of the most common tumour types that are responsible for CNS spread, and included only patients with full clinical follow-up and availability of adequate cranial MRIs at the diagnosis of brain metastasis. We found a strong correlation of TMT with patient prognosis, as each millimetre of baseline TMT reduced the risk of death by 19% in the BC cohort and by 24% in the NSCLC cohort. Importantly, the effect was independent of established prognostic factors. We thus feel that TMT measurement can help to improve the survival prediction of brain metastasis patients in the clinical setting, and may aid in patient selection and stratification in clinical trials.

The temporal muscle was chosen as the muscle of interest for our study because it is one of the few easily measurable craniofacial muscles, which is assessed in its entire expanse on routinely obtained brain MR images. Kilgour et al. [15] suggested the use of cross-sectional areas of neck muscles at the midpoint level of C2 to investigate the age-related loss of muscle mass on brain MR scans. However, a limitation of this technique is that these muscles are, in most cases, only incompletely depicted on routine cranial MR images. In our case series, the neck muscles were not evaluable according to the algorithm of Kilgour et al. in the vast majority of cases. For our study, we used a clear and easily reproducible algorithm to measure TMT with defined anatomical landmarks, and showed excellent inter-rater reliability for this approach. TMT measurement per case took only approximately 30 s per patient, and can, in our experience, easily be incorporated into the clinical workflow.

Our findings are in line with the published literature, as muscle loss has been shown to be a strong adverse prognostic factor in other cancer types, but has, to our knowledge, not been investigated in brain metastasis patients [9, 10]. Cancer-associated cachexia has a multi-factorial aetiology and may be related to insufficient nutrition, catabolic metabolism, paraneoplastic effects, chronic inflammation, anti-neoplastic therapies and other factors [8]. We did not investigate the specific pathobiology of temporal muscle-wasting in our study, but we consider it likely to be reflective of a generalized cancer-associated sarcopenic state. This notion is supported by a previous study that showed a good correlation between skeletal muscle and TMT [13]. Of note, we did not find a correlation between corticosteroid intake at the diagnosis of brain metastasis and TMT. Corticosteroids are known to lead to significant muscle loss over time; however, they are usually prescribed in cases of symptomatic brain oedema in patients with known brain metastases. We measured baseline TMT at the time of brain metastasis diagnosis in our study, and, therefore, patients were unlikely to have been exposed to corticosteroids for a prolonged period of time.

We performed correlations with some patient characteristics and the results of these analyses show that TMT measurement provides information not captured by other clinical parameters. First, we found only a weak inverse correlation of TMT with patient age. This finding indicates that measurement of sarcopenia as an indicator of the patient’s physical condition may be more informative for clinical decision-making than consideration of the chronological age alone. Furthermore, we found no correlation between TMT and BMI. This finding may be explained by the fact that the BMI does not differentiate between lean body mass and fat mass, and TMT measurement is more likely to be sensitive to sarcopenic obesity (low-muscle-obesity) [9]. The fact that the median TMT varies between the NSCLC and the BC patient cohort is based on the difference in gender predominance within these two cohorts. The NSCLC patient cohort consisted of male and female patients in comparison to the BC cohort, which included only female patients. As expected, we observed significantly higher TMT values in male than in female patients. Still, the prognostic role of TMT was independent of patient gender in the NSCLC cohort. Male BC patients are exceedingly rare and were excluded from our study.

Some limitations of our study need to be acknowledged. Due to the retrospective nature of our investigation, we were not able to correlate TMT with muscle strength or actual patient frailty. In order to study such functional-anatomical relationships, prospective studies need to be designed. Furthermore, although a correlation between TMT and skeletal muscle measurements was established in a prior study [13], we were unable to confirm this association in our series. We could not, unfortunately, retrieve a sufficient number of CT scans of the abdomen from our patients, as these had been performed at external institutions in most cases. However, we were able to confirm the prognostic role of TMT in two independent and large patient cohorts, and thus feel that our data provide strong evidence that warrants further investigation of TMT and its clinical applications. The advantage that the temporal muscle is completely depicted on routine MRI scans and is therefore suitable to reflect muscle mass loss on this image modality comes with the disadvantage that it is a relatively small muscle regarding muscle diameter. To increase the measurement accuracy a precise measurement (including image magnification) as well as a strict adherence to the predefined landmarks for TMT measurements are crucial. Further studies may also investigate whether TMT has prognostic relevance in other brain tumour types, including brain metastases from other cancers, as well as primary brain tumours, such as gliomas.

Moreover, patients without a full clinical follow-up including further applied therapies and characteristics of the clinical course were excluded from the analysis to limit the analysis to a rather homogenous cohort of patients with comparable treatment approaches, all primarily treated at our centre. Excluded patients mainly encompassed patients majorly treated at a different centre, especially in nearby countries, who were only transferred to the Medical University of Vienna for the treatment of newly diagnosed brain metastasis. Importantly, fragile patients were therefore more likely to be included in the further analysis as their treatment stayed within our tertiary care centre to adequately address all symptoms. Nevertheless, our data need to be validated in a prospective setting.

The awareness of the relationship between the loss of muscle mass and cancer outcome may lead to new therapeutic targets. Muscle loss in cancer patients is considered to be a multifactorial event involving mainly inflammatory but also catabolic processes. Thus, nutrition alone has not been able to reverse muscle-wasting in cancer patients. Recent trials have focused on the prevention of cancer-related sarcopenia, including exercise training [16], nutritional supplements like omega-3 fatty acids [17] or medication-based concepts such as myostatin inhibitors [18] or melanocortin-4 receptor antagonists [19]. It is, therefore, all the more important to include muscle mass assessment in the routine clinical setting of cancer patients to recognize the onset of muscle mass loss so that interventions to improve or delay the progression of this process can be implemented.

In conclusion, our data show that TMT is a reproducibly assessable parameter that allows survival prediction in patients with brain metastases. TMT may help to better define frail patient populations and may optimize patient selection for therapeutic measures or clinical trials. However, further prospective studies are needed to correlate TMT with other clinical frailty parameters of the patients.

## Electronic supplementary material

Below is the link to the electronic supplementary material.

Supplemental Fig. 1 (DOCX 987 kb)

Supplemental Table 1 (DOCX 18 kb)

## Abbreviations

BC: Breast cancer
BMI: Body mass index
CT: Computed tomography
DS-GPA: Diagnosis-specific graded prognostic assessment
ICC: Intraclass correlations
MRI: Magnetic resonance imaging
NSCLC: Non-small cell lung cancer
OS: Overall survival
TMT: Temporal muscle thickness

## References

[1] Preusser M, Capper D, Ilhan-Mutlu A (2012). Brain metastases: pathobiology and emerging targeted therapies. Acta Neuropathol.

[2] Nayak L, Lee EQ, Wen PY (2012). Epidemiology of brain metastases. Curr Oncol Rep.

[3] Arvold ND, Lee EQ, Mehta MP (2016). Updates in the management of brain metastases. Neuro Oncol.

[4] Berghoff AS, Schur S, Füreder LM (2016). Descriptive statistical analysis of a real life cohort of 2419 patients with brain metastases of solid cancers. ESMO Open.

[5] Sperduto PW, Kased N, Roberge D (2012). Summary report on the graded prognostic assessment: an accurate and facile diagnosis-specific tool to estimate survival for patients with brain metastases. J Clin Oncol.

[6] Kondziolka D, Parry PV, Lunsford LD (2014). The accuracy of predicting survival in individual patients with cancer. J Neurosurg.

[7] Taylor AE, Olver IN, Sivanthan T (1999). Observer error in grading performance status in cancer patients. Support Care Cancer.

[8] Fearon K, Strasser F, Anker SD (2011). Definition and classification of cancer cachexia: an international consensus. Lancet Oncol.

[9] Prado CM, Lieffers JR, McCargar LJ (2008). Prevalence and clinical implications of sarcopenic obesity in patients with solid tumours of the respiratory and gastrointestinal tracts: a population-based study. Lancet Oncol.

[10] Tamandl D, Paireder M, Asari R (2016). Markers of sarcopenia quantified by computed tomography predict adverse long-term outcome in patients with resected oesophageal or gastro-oesophageal junction cancer. Eur Radiol.

[11] Martin L, Birdsell L, MacDonald N (2013). Cancer cachexia in the age of obesity: skeletal muscle depletion is a powerful prognostic factor, independent of body mass index. J Clin Oncol.

[12] Jones KI, Doleman B, Scott S (2015). Simple psoas cross-sectional area measurement is a quick and easy method to assess sarcopenia and predicts major surgical complications. Color Dis.

[13] Ranganathan K, Terjimanian M, Lisiecki J (2014). Temporalis muscle morphomics: the psoas of the craniofacial skeleton. J Surg Res.

[14] Bender R, Lange S (2001). Adjusting for multiple testing - when and how?. J Clin Epidemiol.

[15] Kilgour AHM, Subedi D, Gray CD (2012). Design and validation of a novel method to measure cross-sectional area of neck muscles included during routine mr brain volume imaging. PLoS One.

[16] Argilés JM, Busquets S, López-Soriano FJ (2012). Are there any benefits of exercise training in cancer cachexia?. J Cachex Sarcopenia Muscle.

[17] Di Girolamo FG, Situlin R, Mazzucco S (2014). Omega-3 fatty acids and protein metabolism: enhancement of anabolic interventions for sarcopenia. Curr Opin Clin Nutr Metab Care.

[18] Padhi D, Higano CS, Shore ND (2014). Pharmacological inhibition of myostatin and changes in lean body mass and lower extremity muscle size in patients receiving androgen deprivation therapy for prostate cancer. J Clin Endocrinol Metab.

[19] Dallmann R, Weyermann P, Anklin C (2011). The orally active melanocortin-4 receptor antagonist BL-6020/979: a promising candidate for the treatment of cancer cachexia. J Cachex Sarcopenia Muscle.

