# Supplementary material for: Survival prediction using temporal muscle thickness measurements on cranial magnetic resonance images in patients with newly diagnosed brain metastases
Source: Eur Radiol. 2017 Jan 3;27(8):3167–73. doi: 10.1007/s00330-016-4707-6 (PMC5491578; doi:10.1007/s00330-016-4707-6)
Supplement: Supplementary file 1 — (DOCX 987 kb) [file 330_2016_4707_MOESM1_ESM.docx]

**
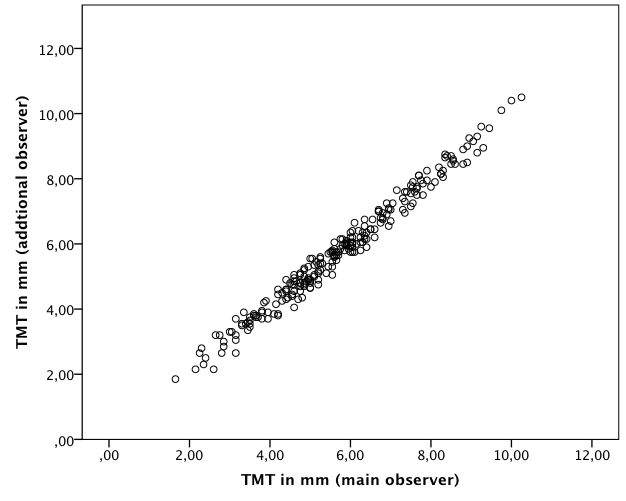
**

**Supplemental Figure 1**: Correlation of TMT as assessed by the main observer and the additional observers.
