# Supplementary material for: Survival prediction using temporal muscle thickness measurements on cranial magnetic resonance images in patients with newly diagnosed brain metastases
Source: Eur Radiol. 2017 Jan 3;27(8):3167–73. doi: 10.1007/s00330-016-4707-6 (PMC5491578; doi:10.1007/s00330-016-4707-6)
Supplement: Supplementary file 2 — (DOCX 18 kb) [file 330_2016_4707_MOESM2_ESM.docx]

**Table 1:** Patient Characteristics

| **Table 1A**: Patient characteristics in the BC cohort | | |
| --- | --- | --- |
|  | **BC cohort (n=188)** | |
|  | n | % |
| Median age at diagnosis of brain metastasis, years  (range) | 54  (30-85) | |
| Gender |  |  |
| Female | 188 | 100.0 |
| Breast cancer subtype |  |  |
| Luminal A | 45 | 23.9 |
| HER2 | 75 | 39.9 |
| Triple negative | 34 | 18.1 |
| Unknown | 34 | 18.1 |
| Median time from diagnosis of primary tumor to diagnosis of brain metastasis, months  (range) | 43.5  (-1 – 443) | |
| Median BMI  (range) | 25.5  (18.0-38.0) | |
| Cortisone treatment |  |  |
| Yes | 94 | 50.0 |
| No | 49 | 26.1 |
| Unknown | 45 | 23.9 |
| Diagnosis-specific GPA |  |  |
| 0-1.0 | 6 | 3.2 |
| 1.5-2.0 | 44 | 23.4 |
| 2.5-3.0 | 61 | 32.4 |
| 3.5-4.0 | 43 | 22.9 |
| Unknown | 34 | 18.1 |
| 1^st^ line treatment of brain metastasis |  |  |
| SRS | 77 | 41.0 |
| Chemotherapy | 2 | 1.1 |
| Neurosurgical resection | 48 | 25.5 |
| WBRT | 60 | 31.9 |
| Best supportive care | 1 | 0.5 |
| Alive at last follow-up |  |  |
| Yes | 15 | 8.0 |
| No | 173 | 92.0 |
| Median overall survival from diagnosis of brain metastasis, months  (range) | 10  (0-91) | |
|  | | |
| **Table 1B**: Patient characteristics in the NSCLC cohort | | |
|  | **NSCLC cohort (n=247)** | |
|  | n | % |
| Median age at diagnosis of brain metastasis, years  (range) | 62  (33-88) | |
| Gender |  |  |
| Male | 139 | 56.3 |
| Female | 108 | 43.7 |
| Median time from diagnosis of primary tumor to diagnosis of brain metastasis, months  (range) | 31  (-7 – 104) | |
| Median BMI  (range) | 24.0  (15.0-44-0) | |
| Cortisone treatment |  |  |
| Yes | 170 | 68.8 |
| No | 64 | 25.9 |
| Unknown | 13 | 5.3 |
| Diagnosis specific GPA |  |  |
| 0-1.0 | 66 | 26.7 |
| 1.5-2.0 | 108 | 43.7 |
| 2.5-3.0 | 61 | 24.7 |
| 3.5-4.0 | 12 | 4.9 |
| 1^st^ line treatment of BM |  |  |
| SRS | 119 | 48.2 |
| Chemotherapy | 5 | 2.0 |
| Neurosurgical resection | 72 | 29.1 |
| WBRT | 51 | 20.7 |
| Best supportive care | 0 | 0.0 |
| Alive at last follow-up |  |  |
| Yes | 27 | 10.9 |
| No | 220 | 89.1 |
| Median overall survival from diagnosis of BM, months  (range) | 8  (0-77) | |
